# Preoperative assessment of mitral valve abnormalities in left atrial myxoma patients using cardiac CT

**DOI:** 10.18632/oncotarget.16139

**Published:** 2017-03-11

**Authors:** Jing Chen, Zhi-Gang Yang, En-Sen Ma, Qin Zhang, Xi Liu, Ying-Kun Guo

**Affiliations:** ^1^ Department of Radiology, West China Hospital, Sichuan University, Chengdu, Sichuan, China; ^2^ Department of Radiology, West China Second University Hospital, Sichuan University, China; ^3^ Department of Radiology, The Affiliated Hospital of Southwest Medical University, Luzhou, Sichuan, China; ^4^ Department of Radiology, China–Japan Friendship Hospital, Yinghua Dongjie, Chaoyang, Beijing, China

**Keywords:** cardiac myxoma, dual-source CT, mitral valve, mitral regurgitation, mitral stenosis

## Abstract

**Background:**

To retrospectively evaluate mitral valve abnormality in left atrial myxoma patients by using cardiac computed tomography (CT).

**Material and methods:**

Cardiac CT was performed in 56 patients with left atrial myxoma and 50 controls. Tumor and mitral valve characteristics were analyzed. The mitral valve parameters differences were compared between patients with myxoma and controls, myxoma with or without mitral valve obstruction, different obstruction degrees, respectively. Receiver operating characteristic analysis was performed to determine the cut-off values of abnormal mitral valve parameters for myxoma patients. Multiple linear regression, logistic regression models and cox regression analysis were used to determine factors associated with mitral valve abnormalities, mitral obstruction, mitral regurgitation and postoperative recovery, respectively.

**Results:**

Myxoma induced the dilation of mitral valve, with different results among different degrees of obstruction (*p*<0.001). Mitral valve parameters had relationship with myxoma parameters. The cut-off values for discriminating mitral valve abnormalities in myxoma patients were found. Some significant predictors for mitral obstruction were tumor pedicle-tumor volume and patient age (HR, 0.886-30.811; *p* = 0.011-0.043). Moreover, the predictor for mitral regurgitation was mitral annulus diameter in diastolic phase (HR, 20.862; 95%CI,1.331-327.100; *p* = 0.031). Some predictors associated with postoperative recovery of mitral regurgitation were age, mitral annulus area, mitral annulus diameter and mitral valve diameter cutoff value for diastolic phase (HR, 0.001-119.160; *p* = 0.012-0.028).

**Conclusion:**

Cardiac CT is capable of quantitatively assessing myxoma characteristic and mitral valve abnormality induced by myxoma, thus providing guidance of operative management and postoperative evaluation.

## INTRODUCTION

Cardiac myxoma (CM) is the most prevalent type of primary cardiac tumor, comprising more than 50% of the total [1]. It originates from any chamber of the heart, but most occur in the left atrial septum [2]. A CM could induce relative mitral stenosis or regurgitation from back and forth movement in the left chamber during the cardiac cycle, and even lead to severe hemodynamic abnormalities with the risk of postural syncope, embolism, and sudden death [3]. Therefore, CM should be treated actively to prevent life-threatening complications, and as soon as possible to restore heart function. If the heart valves of myxoma patients are damaged seriously, surgical heart valve repair or replacement is necessary [4–9]. Thus, an accurate and non-invasive imaging modality for evaluating these anatomic features before surgery is imperative.

CT could simultaneously observe the tumor characteristics, coronary artery disease, and cardiac function, providing better guidance for comprehensive clinical evaluation and surgical treatment. Moreover, it can clearly show and evaluate the severity of valvular disease and measure the hemodynamic parameters of the cardiac cycle in a semi-quantitative manner [10–13], which could be used as a favorable supplement to ultrasound. Data from previous studies mainly focused on the application value of imaging methods for diagnosis of and noting the anatomical characteristics of cardiac myxoma [3, 14–18]. However, these studies could not comprehensively assess the preoperative feasibility and guide surgical decision for cardiac myxoma, because data on the influence of cardiac structure by myxoma are lacking, especially concerning the mitral valve. So far, the assessment of mitral valve abnormalities in left atrial myxoma patients are not sufficiently clarified owing to the relatively few number of populations [4, 8, 19–21]. Moreover, simultaneous evaluations of myxoma and mitral valve in left atrial myxoma patients are also limited. Thus, the purpose of this study was to further describe and evaluate the mitral valve abnormalities caused by left atrial myxomas in a relatively larger population using cardiac CT for making surgical decision prior to surgery.

## MATERIALS AND METHODS

### Study population

This study was conducted in accordance with the Declaration of Helsinki (2000 Edition), and an application for the exemption of patients’ informed consent was approved by the Institutional Review Board of our hospital, due to the retrospective nature of the study.

In our study, 106 consecutive subjects who underwent cardiac CT were enrolled, including 56 patients with left atrial myxoma and 50 matched normal control subjects. From March 2012 to September 2016, sixty patients with left atrial myxoma who were admitted for surgery at our hospital were enrolled in this study. Patients with a pathologic diagnosis of myxoma after surgery were included. Exclusion criteria included patients with a left ventricular ejection fraction of less than 40%, renal dysfunction (glomerular filtration rate < 30 mL/min/1.7 m2), severe heart failure, known allergy to iodine-based contrast medium, and inadequate image quality for imaging analysis. Fifty patients with suspected coronary disease who underwent cardiac CT during the same period but were finally diagnosed with a normal condition served as the control group. The exclusion criteria of the control group were the patients with inadequate image quality for imaging analysis. After applying the exclusion criteria, 56 patients with left atrial myxoma remained. A control group of 50 age- and gender-matched patients with adequate images was also kept.

### Cardiac CT technique

Dual-source CT (Somatom Definition, Siemens Medical Solutions, Germany) scanning was performed for all patients in a cranio-caudal direction with a retrospective ECG-gated mode. Scanning parameters were as follows: gantry rotation time 330 ms, collimation was 64×0. 6 mm, tube potential (weight ≥ 85 kg, 120 kV for each tube; weight < 85 kg, 100 kV for each tube); and tube current (BMI ≥ 25, 330 mAs per rotation; BMI < 25, 220 mAs per rotation). Only 60-80 mL of non-ionic contrast agent (iopamidol, 370 mg/mL; Bracco Sine Pharmaceutical Corp. Ltd, Shanghai, China) was injected at a flow rate of 5.0 mL/s, followed by a saline flush of 20 mL at the same flow rate. Once the contrast concentration of the interest region on the left atrium reached the threshold of 100-HU, the image acquisition was automatically initiated after a 5-second delay.

### Imaging analysis

Two experienced radiologists, who were unaware of the final clinical diagnosis, independently reviewed each CT image on a dedicated workstation (Leonardo, Siemens Medical Systems). Any discrepancies of anatomical variants and measurements between interpretations were resolved by discussion until a consensus was reached. All data were loaded into three-dimensional (3D) cardiac post-processing software. Multiplanar reformations (MPR), maximum intensity projection (MIP), and volume rendered technique (VRT) with 3D or discretionary directions images were used for observing left atrial myxoma and the mitral valve.

**Figure 1:**
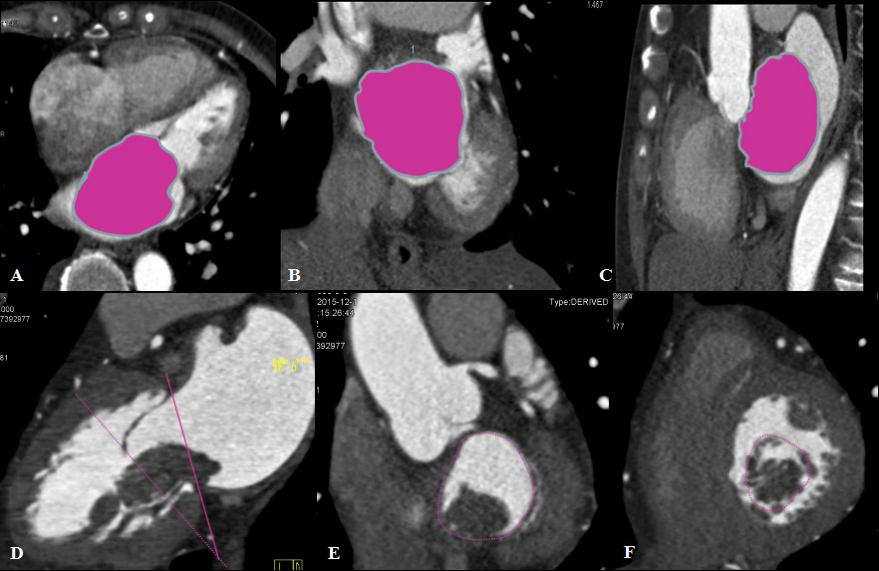
Measurements of tumor volume and mitral valve perimeters Multiplanar reconstruction showed the measurement of tumor volume (red color) in transverse, coronal, and sagittal planes **A.**-**C.** The short axis plane of mitral annulus **E.** was obtained paralleling to mitral annular plane (red line) in the standard long axis view of left ventricle **D.** In addition, the short axis plane of the mitral valve opening **F.** is shown paralleling the mitral valve leaflet plane (red dotted line) in the standard long axis view of the left ventricle.

The parameters of left atrial myxoma included tumor size, location, range of motion, pedicle, and obstruction condition. Among them, the size indexes consisted of whole volume, diameter, and area at the level of mitral annulus while the mitral valve opening was in end-diastole phase. The tumor volume was obtained on the relative post-processing software of CT (Figure 1A-1C). The location of the tumor was divided into upper and lower regions by the mid-level of the left atrium. Left atrial myxoma with mitral valve obstruction was defined as the tumor prolapsed into the level of mitral annulus and even into the left ventricle during diastole phase, and the degree of obstruction was divided into three levels according to the ratio of tumor area to mitral annulus area. As is shown in Figure 1, mitral annular diameter (MAD) and mitral valve diameter (MVD) were measured in standard long axis view of the left ventricle (Figure 1D). Mitral annular area (MAA), mitral annular perimeter (MAP), and mitral valve area (MVA) were measured in standard short axis view of left ventricle (Figure 1E-1F). All the measurements of mitral valve size were performed in both of end-diastolic and end-systolic phases. Left atrial diameters, including maximum transverse, anteroposterior, and length, were measured at different levels of the left atrium. And, left ventricular diameter was measured in long axis view of the left ventricle [22]. All the measurements were performed twice by two independent researchers. The anteroposterior diameter of mitral annulus > 3.5 cm conventionally is considered dilated [23].

### Statistical analysis

All statistical analyses were performed using dedicated software (SPSS, Version 16; Chicago, IL, US). Dichotomous data were expressed as numbers and percentages, and continuous data were recorded as mean values and corresponding standard deviations. Paired group comparisons were performed using paired t test. Receiver operating characteristic (ROC) analysis was performed to determine whether the cut-off values of mitral valve parameters could be used to differentiate mitral valve perimeter abnormalities between myxoma patients and normal subjects. To determine factors associated with sizes of mitral valve, mitral abnormalities, or postoperative recovery in myxoma patients, Spearman rank correlation was developed to obtain the coefficient, and multiple linear regression, logistic regression models, and cox regression analysis were used to calculate hazard ratios with 95% CI. Covariates included tumor size, location, pedicle, obstruction degrees, mitral regurgitation, tumor diameter or area at the level of mitral annulus and mitral valve opening, mitral valve size, and their cut-off value. Differences with p-values less than 0.05 were considered significant.

## RESULTS

### Preoperative baseline characteristics

DSCT scanning was successfully performed in all 106 subjects, and mitral valves and tumors were clearly shown on the images. The effective radiation dose for patients were 3.5±1.3mSv. Baseline characteristics of patients in our study are listed in Table 1. Of the basic clinical data, there was no significant difference between the myxoma patients and normal controls, except that the left ventricular ejection fraction decreased in the myxoma patients group (*p* = 0.031). Preoperative cardiac function classifications (NYHA) of all 56 patients were composed of three cases with grade I, 17 with grade II, and 15 with grade III. Three patients, including two patients with cerebral infarction and one with myocardial infarction, were cured before surgical resection of left atrial myxoma. In addition, eight patients opted for mitral annuloplasty or replacement over myxoma resection. Coronary artery bypass grafting was also done in one of these patients.

**Table 1:** Baseline characteristics of left atrial myxoma patients and control individuals

	Left atrial myxoma (*N*= 56)	controls (*N*= 50)	*p* value	
Age, y	64±10	61±9	0.152
Male gender (male:female)	22:34	20:30	0.940
Weight, Kg	59.3±9.1	58.1±9.7	0.064
BMI, kg/m2	21.9±2.5	21.5±3.1	0.521
Blood pressure, mm Hg	117.2±19.4/79.3±9.5	113.4±19.7/80.9±9.9	0.636
Heart rate, bpm	80.5±14.9	78.9±11.0	0.402
LVEF, %	56.1±11.7	59.6±7.3	0.031
**Concomitant heart diseases**			
Coronary artery diseases	13	-	-
pericardial effusion	2	-	-

Abbreviations: BMI, body mass index; LVEF, left ventricular ejection fraction.

### CT manifestations of tumors

**Figure 2:**
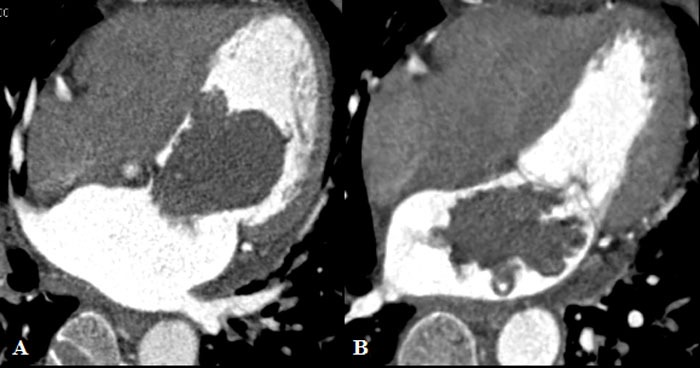
The shape of myxoma in different cardiac cycles Tumor morphology was regular in shape in the diastolic phase **A.**, and irregular in shape in the systolic phase **B.**

In total, 42 patients had tumor pedicle, 0.6-1.5 cm in length. All the tumors were solitary, with measurements of 3.1±1.0 cm in diameter and 32.6±28.4 cm3 in tumor volume for the diastole phase. The locations of tumor were classified into the following two regions: 19 subjects above the mid-level of the left atrium, 37 patients below the mid-level. Myxoma densities in CT image were 24.1±8.6 HU for plain and 79.6±20.5 HU for enhancement. Calcification was found in nine patients. Commonly, tumor morphology was regular in the diastolic phase, and irregularly shaped in the systolic phase (Figure 2). Tumor diameter and area at the level of mitral annulus were 2.6±0.8 cm and 5.6±2.9 cm2, and in the mitral orifice plane they were 2.7±0.9 cm and 5.5±4.3 cm2.

### CT manifestations of mitral valve in left atrial myxoma patients

In all 56 patients, mitral annulus enlargements were totally detected in 35 (62.5%) cases. The mitral valve sizes in left atrial myxoma patients were larger than those of control subjects in both the end-diastolic and end-systolic phases (all *p* < 0.050). The diameters of left atrium in the myxoma group were larger than those of the control subjects (*p* < 0.001), except for anteroposterior diameter (*p* = 0.240). More importantly, the mitral valve sizes of left atrial myxoma patients with mitral valve obstruction were larger than those patients without obstruction (*p* < 0.050), apart from MAP and MAD in the end-systolic phase (*p* = 0.070, *p* = 0.187) (Table 2). No significant differences of left cardiac sizes were found between patients with and without mitral valve obstruction.

**Table 2:** Measurement of mitral valve parameters between different groups

		Myxoma (*N* = 56)	Controls (*N*= 50)	*p* value	Myxoma with obstruction (*N*= 37)	Myxoma without obstruction (*N*= 19)	*p* value
MAA (cm2)	End-diastolic phase	9.7±2.6	8.4 ±1.3	0.001	10.1±2.5	7.7±2.6	0.002
	End-systolic phase	8.3± 2.2	6.1±1.1	<0.001	8.8±2.3	7.3±1.6	0.023
MAP (cm)	End-diastolic phase	11.3±1.8	10.6±0.8	0.013	11.6±1.9	10.6±1.2	0.039
	End-systolic phase	11.1±1.3	10.2±1.2	0.001	11.4±1.3	10.6±1.2	0.070
MAD (cm)	End-diastolic phase	3.6±0.6	3.3±0.3	0.015	3.7±0.5	3.5±0.5	0.044
	End-systolic phase	3.5± 0.4	3.1± 0.3	<0.001	3.6±0.4	3.4±0.4	0.187
MVA (cm2)	End-diastolic phase	5.5± 2.9	3.6± 0.6	<0.001	6.3±3.1	3.8±1.1	<0.001
MVD (cm)	End-diastolic phase	2.6± 0.7	2.3± 0.2	0.015	2.8±0.7	2.1±0.2	<0.001

The parameters were expressed as mean values ± standard deviations.

Abbreviations: MAA, mitral annulus area; MAP, mitral annulus perimeter; MAD, mitral annulus diameter; MVA, mital valve area; MVD, mitral valve diameter.

Myxoma with mitral valve obstruction was found in 37 cases (66.1%), including mild (7 cases), moderate (18 cases), and severe patients (12 cases). All the mitral valve parameters were different among the three groups with different severity of obstruction (all *p* < 0.001) (Table 3), especially severe obstruction. And the mitral valve parameters in myxoma patients with severe mitral obstruction were significantly larger than those in patients with mild or moderate obstruction (*p* < 0.001). But, little significant difference for mitral valve sizes was found between patients with mild and moderate mitral valve obstruction (*p* < 0.050).

**Table 3:** Measurement of mitral valve parameters among different mitral valve obstruction groups in left atrial myxoma patients

		Mild (*N* = 7)	Moderate (*N* = 18)	Severe (*N* = 12)	*p* value
MAA (cm2)	End-diastolic phase	8.2±1.0	9.6±1.5	13.0±2.7	<0.001
	End-systolic phase	7.2±0.6	8.1±1.7	11.4±1.7	<0.001
MAP (cm)	End-diastolic phase	10.5±0.6	10.9±0.8	13.4±2.3	<0.001
	End-systolic phase	10.5±0.6	10.9±0.8	12.8±1.2	<0.001
MAD (cm)	End-diastolic phase	3.5±0.2	3.6±0.4	4.2±0.4	<0.001
	End-systolic phase	3.3±0.2	3.4±0.4	4.0±0.4	<0.001
MVA (cm2)	End-diastolic phase	4.5±1.0	5.3±1.5	9.0±4.0	<0.001
MVD (cm)	End-diastolic phase	2.3±0.2	2.6±0.6	3.4±0.7	<0.001

The parameters were expressed as mean values ± standard deviations. The abbreviations are the same as in Table 2

On echocardiography, left atrial myxoma patients with mitral regurgitation were detected in 22 cases including mild (18 cases), moderate (3 cases), and severe patients (1 cases), and mitral stenosis was observed in 16 cases. At 3 months’ follow-up after the myxoma was removed, all the cases of mitral stenosis had been corrected, whereas there were still 15 patients (14 mild and 1 moderate cases) with non-improvement mitral regurgitation.

### Correlations between myxoma and mitral valve abnormalities

The correlations of left atrial myxoma and mitral valve parameters are summarized and shown in Table 4. MAA and MAD correlated well with tumor diameter, mitral valve obstruction, and tumor area at the level of mitral annulus (r = 0.046-0.638; *p* < 0.050). MAP was related to mitral valve obstruction and tumor area at the level of mitral annulus (r = 0.793, 0.248; *p* < 0.010). MVA and MVD were related to tumor area at the level of mitral annulus and the level of mitral valve opening (r = 0.042-0.226; *p* < 0.050). Moreover, MVA correlated with tumor diameter at the level of mitral valve opening (r = 0.494; *p* < 0.029).

**Table 4:** Multiple linear regression analysis of multiple risk factors and mitral valve parameters

	variable	B	S.E	*t*	*p*
	TD	0.104	0.049	2.104	0.041
**MAD**	Obstruction	−0.513	0.162	−3.170	0.003
	TAMA	0.150	0.046	3.260	0.002
	TD	0.420	0.195	2.158	0.036
**MAA**	Obstruction	−1.568	0.638	−2.458	0.018
	TAMA	1.113	0.182	6.121	<0.001
	Obstruction	−2.929	0.793	−3.694	0.001
**MAP**	TAMA	1.012	0.248	4.082	<0.001
	TAMA	0.544	0.226	2.412	0.020
**MVA**	TAMVO	0.775	0.190	4.077	<0.001
	TDMVO	−1.114	0.494	−2.255	0.029
	TAMA	0.131	0.050	2.608	0.012
**MVD**	TAMVO	0.134	0.042	3.151	0.003

Abbreviations: TD, tumor diameter; TAMA, Tumor area at the level of mitral annulus; TAMVO, Tumor area at the level of mitral valve opening; TDMVO, Tumor diameter at the level of mitral valve opening. Other abbreviations are the same as in Table 2.

**Figure 3:**
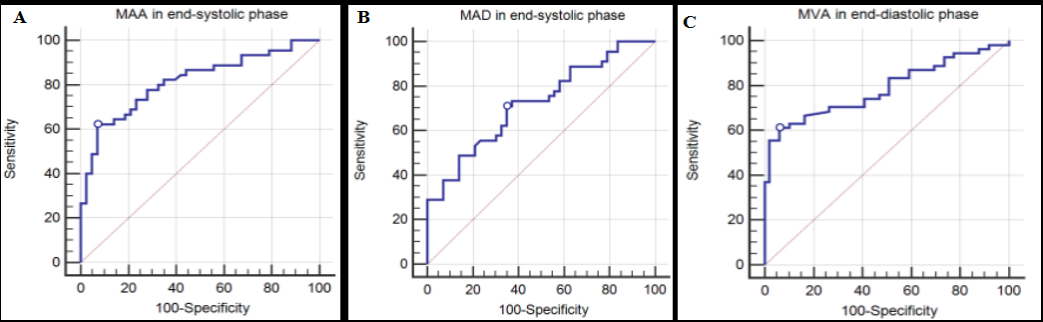
Receiver operating characteristic (ROC) analysis of abnormal mitral valve parameters The cut-off values for mitral annulus area (MAA), mitral annulus diameter (MAD), and mitral valve area (MVA) assisted in discriminating mitral valve abnormalities in left atrial myxoma patients **A.**-**C.**

**Figure 4:**
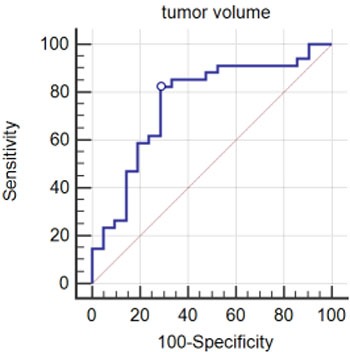
Receiver operating characteristic (ROC) analysis of tumor volume for dilated mitral annulus The cut-off value was 14.92 cm3, sensitivity was 82.4, and specificity was 71.4. The area under the curve was 0.758.

Following ROC analysis of the mitral valve parameters, we found that the cut-off values for them assisted in discriminating between left atrial myxoma patients and normal controls, especially MAA, MAD, and MVA (Figure 3). The area under the ROC curve, and the sensitivity and specificity of the aforementioned data used for the discrimination of left atrial myxoma patients and normal controls are summarized in Table 5. In addition, we found the cut-off value for tumor volume caused the enlargement of mitral annulus (Figure 4). The cut-off value, the area under the ROC curve, and the sensitivity and specificity were 14.92 cm3, 0.758, 82.4%, and 71.4%, respectively (*p* < 0.001).

**Table 5:** ROC analysis of mitral valve parameters for detecting initial abnormalities between left myxoma patients and normal controls

		Cutoff	AUC	Sensitivity (%)	Specificity (%)	*p*
MAA (cm2)	End-diastolic phase	10.1	0.661	40.0	94.1	0.028
	End-systolic phase	7.37	0.816	62.2	93.0	<0.001
MAP (cm)	End-diastolic phase	11.6	0.614	32.7	94.1	0.037
	End-systolic phase	10.62	0.692	62.2	69.8	0.001
MAD (cm)	End-diastolic phase	3.7	0.698	41.8	94.1	<0.001
	End-systolic phase	3.22	0.722	71.1	65.1	<0.001
MVA (cm2)	End-diastolic phase	4.2	0.786	61.1	93.9	<0.001
MVD (cm)	End-diastolic phase	2.8	0.561	31.5	100.0	0.303

Abbreviations: ROC, Receiver Operating Characteristic; AUC, area under the ROC curve. Other abbreviations are the same as in Table 2.

Some significant predictors of mitral obstruction were tumor pedicle, tumor volume, and patient age (HR, 0.886-30.811; *p* = 0.011-0.043) (Table 6). Moreover, MAD in the diastolic phase was the significant predictor of mitral regurgitation (HR, 20.862; 95%CI, 1.331-127.100; *p* = 0.031). There were no differences in outcome among other factors (all *p* < 0.05) (Table 6).

**Table 6:** Hazard Ratios for All-Cause mitral abnormalities or postoperative recovery in Logistic and Cox regression analyses

		Cox analysis				Logistic analysis	
	Postoperative recovery			Mitral obstruction		Mitral regurgitation	
Variable	*P* Value	Hazard Ratio (95% CI)	*p* Value		Hazard Ratio (95% CI)	*P* Value	Hazard Ratio (95% CI)
Age	**0.012**	1.328 (1.063-1.658)	**0.043**		0.886(0.788-0.996)		
Sex	0.164	0.130 (0.007-2.304)	0.785		0.771(0.120-4.978)		
Location	0.181	0.053 (0.001-3.902)	0.076		0.129(0.013-1.239)	0.508	1.741(0.338-8.979)
Tumor pedicle	0.462	0.378 (0.028-5.052)	**0.017**		30.811(1.849-113.506)	0.911	1.098(0.214-5.639)
Tumor volume	0.107	0.944 (0.881-1.012)	**0.011**		1.098(1.022-1.181)	0.234	0.979(0.946-1.014)
MAACO in end-diastolic phase	**0.028**	19.160 (1.674-80.942)	0.407		4.884(0.115-207.095)		
MAPCO in end-diastolic phase	0.187	14.151 (0.277-73.481)	0.825		0.646(0.013-31.296)		
MADCO in end-diastolic phase	**0.018**	0.035 (0.002-0.570)	0.159		6.973(0.468-103.867)		
MVACO	0.584	0.421 (0.019-9.274)	0.243		0.259(0.027-2.507)		
MVDCO	**0.022**	0.001 (0.000-0.340)	0.227		0.116(0.004-3.816)		
Mitral obstruction	0.102				0.372	2.093 (0.414-10.595)
-mild	**0.037**	61.863 (1.275-302.207)				
-moderate	**0.016**	65.182 (1.695-340.682)				
-severe	0.058	87.743 (0.804-597.049)				
MAA in end-systolic phase					0.453	1.303 (0.653-2.598)
Dilated mitral annulus	0.140	16.868 (0.394-71.493)			0.947	0.950 (0.211-4.284)
MAP in end-systolic phase					0.465	0.741 (0.332-1.655)
MAD in end-systolic phase					**0.031**	20.862 (1.331-127.100)
Mitral regurgitation	0.764					
-mild	0.318	0.264 (0.019-3.593)				
-moderate	0.939	0.807 (0.003-189.761)				
-severe	0.998	0.043 (0.000-1.231)				
TAMVO	0.070	3.313 (0.906-12.118)				
TDMVO	0.057	0.030 (0.001-1.109)				
Mitral valve surgery	0.159	0.008 (0.000-6.634)				

Abbreviations: MAACO, mitral annulus area cut-off value; MAPCO, mitral annulus perimeter cut-off value; MADCO, mitral annulus diameter cut-off value; MVACO, mitral valve area cut-off value; MVDCO, mitral valve diameter cut-off value; TAMVO, tumor area at the level of mitral valve opening; TDMVO, tumor diameter at the level of mitral valve opening. Other abbreviations are the same as in Table 2. All the above cut-off valves were obtained by using ROC analysis.

Additional significant univariable predictors of postoperative recovery for mitral regurgitation are listed in Table 6. Some covariables that were found to be independently associated with postoperative recovery of mitral regurgitation in the multivariable model were age (HR, 1.328; 95% CI, 1.063-1.658; *p* = 0.012), MAA, MAD, and MVD cut-off value for the diastolic phase (HR, 19.160, 0.035, 0.001; *p* = 0.028, 0.018, 0.022), and the cut-off values were obtained according to the ROC results. In the multivariable Cox regression model, patients with mild mitral obstruction (HR, 61.863; 95% CI, 1.275-302.207; *p* = 0.037) or moderate mitral obstruction (HR, 65.182; 95% CI, 1.695-340.682; *p* = 0.016) had a significantly higher risk of postoperative recovery of mitral regurgitation in comparison with those with no mitral obstruction (Table 6).

### Inter-operator reproducibility

Intraobserver intraclass correlation coefficient (ICC) was 0.996 for MAA and MAP in the end-diastolic phase, respectively. ICC was 0.985 for MAD, 0.998 for MVA, and 0.992 for MVD. Moreover, in the end-systolic phase, ICC was 0.998 for MAA, 0.996 for MAP, and 0.986 for MAD. The interobserver ICC was 0.958 for MAA end-diastolic phase, 0.961 for MAP, 0.986 for MAD and MVD, 0.955 for MVA.

## DISCUSSION

### Main findings

So far, This is the first timeto assess mitral valve characteristics of left atrial myxoma patients with a relatively larger sample size, providing more information about the mitral valve abnormalities by myxoma. We found that left atrial myxoma was prone to enlarge the mitral annulus and induce the mitral obstruction/regurgitation. Moreover, the grievously damaged patients not only underwent conventional myxoma resection, but also underwent valve repair or replacement (which, in accordance with the traditional additional mitral valve surgery standard, did not improve the postoperative recovery of mitral regurgitation). Because some predictors were related to the postoperative recovery, additional mitral valve surgery may be more popular for these myxoma patients. In addition, simultaneous evaluating of myxoma and mitral valve in left atrial myxoma patients using DSCT is limited. In clinical practice, comprehensive evaluation could provide an anatomical road map to determine surgical strategy.

### Myxoma characteristics

In our study, patients with CM ranged in age from 35 to 80 years, with a mean age of 64 years, which is slightly higher than the ages in previous reports [24–25]. CM is more common in women [26], which may be related to female hormones [27]. However, patients with CM lack sex hormone receptor expression; thus, the high proportion of CM in women remains to be further studied. As shown in previous reports, left atrial myxoma commonly occurred in the fossa ovalis, and most of them had a pedicle attached to the atrial septum, which is in line with our study. It is rare that a rupture of the tumor pedicle leads to embolism. The tumor usually moves during the cardiac cycle, and the shape of tumor often changes following blood flow, gone with the “wind” and “seaweed” sign. The tumor can easily fall off due to loose tissue structure, leading to embolism. Therefore, it should be operated on as soon as possible to avoid the occurrence of life-threatening complications and restore cardiac function.

### Mitral valve information

In our series, left atrial myxoma can cause mitral annulus enlargement, and mitral valve parameters were different between different degrees of obstruction, especially severe obstruction, but the reason for this is unclear. Some reports proposed that a relatively larger myxomatous mass moved back and forth from the left atrium and left ventricle during the systolic and diastolic phases. Thus, the tumor has been mechanically stretching the mitral annulus in each cardiac cycle, resulting in enlargement [28]. However, this theory could not fully explain our findings, with the differences for mitral valve size between obstruction and non-obstruction, or the different degrees of obstruction. We thought that tumor obstructed the mitral annulus and the valve opening level, resulting in a reduction of relative area. To serve the normal supply, the mitral annulus and mitral valve opening compensated by expansion to get more blood flow. As reported, more than half of the left atrial myxomas have obstruction symptoms [29], of which only 10% can cause serious mitral stenosis [30]. In this group, the data is higher than in other reports. This is because the mitral valve obstruction in this group was the image feature. Although the image showed the obstruction, there was no clinical symptom of obstruction. This type of patient may have mechanical damage to the mitral valve leaflet and the supporting structure (e.g., tendon) followed by the long-term movement of the tumor pedicle, resulting in mitral valve obstruction [31]. In addition, tumor volume was the predictor of mitral obstruction. Larger tumors could easily obstruct the mitral valve. Whether the tumor located where, if the tumor volume up to some threshold value, the mitral annulus could enlarge and get to the further deterioration. Therefore, left atrial myxoma should be treated early. In this article, we could report valuable data concerning enlarged mitral annulus and mitral valve opening. Compared with anteroposterior diameter criteria for dilated mitral annulus, our data may be more comprehensive.

In this series, mitral regurgitation occurred in 22 (39.3%) patients, which is related to MAD in the diastolic phase. This may be the larger diameter of the mitral annulus needed to induce the closure of the valve opening which is attached to the valve ring, resulting in the reverse flow.

Moreover, we found that the traditional additional mitral valve surgery standard could not improve the postoperative recovery of mitral regurgitation. Patients with larger than the threshold of diastolic MAA ( > 10.1 cm2) had poor postoperative recovery. And patients with mild and moderate mitral obstruction recovered poorly compared with patients with no obstruction. That is, if the left atrial myxoma patients with larger MAA and/or mild-to-moderate mitral valve obstruction undergo additional mitral valve surgery, there may be a better postoperative recovery of valve regurgitation.

## LIMITATIONS

This study is not free from limitations. First, the sample size is relatively small, due to less incidence of cardiac myxoma. Second, a limitation of the use of CT is the radiation dose, whereas radiation exposure from DSCT with attenuation-based online tube current modulation technique is reduced by 60% when compared with single-source CT (approximately 8 mSv). Furthermore, CT has a wider availability to overcome the MR contraindications and echocardiography limitations for providing more preoperative information of left atria myxoma. Third, in our study, long-term follow-up data were lacking. We want to present these data in a future study.

In conclusion, left atrial myxoma could enlarge the mitral annulus and induce mitral obstruction/regurgitation. Moreover, the traditional additional mitral valve surgery standard may not improve the postoperative recovery of mitral regurgitation. Some predictors were related to the postoperative recovery. Therefore, these patients may choose to have additional mitral valve surgery for better postoperative recovery. DSCT is capable of quantitatively assessing myxoma characteristics and mitral valve abnormality induced by myxoma, which could be used as a favorable supplement to ultrasound, thus providing guidance for surgical management and postoperative evaluation.

## Abbreviations

DSCT: Dual-source computed tomography
ROC: Receiver Operating Characteristic
MAA: mitral annulus area
MAP: mitral annulus perimeter
MAD: mitral annulus diameter
MVA: mital valve area
MVD: mitral valve diameter
CM: cardiac myxoma
MPR: Multiplanar reformations
MIP: maximum intensity projection
VRT: volume rendered technique
ICC: Intraobserver intraclass correlation coefficient.
